# Supplementary material for: Construction of competing endogenous RNA interaction network as prognostic markers in metastatic melanoma
Source: PeerJ. 2021 Sep 15;9:e12143. doi: 10.7717/peerj.12143 (PMC8449535; doi:10.7717/peerj.12143)
Supplement: Supplemental Information 1 [file peerj-09-12143-s001.docx]

**Supplementary table 1. Sequences of RP11-594N15.3 probes**

| **Gene name** | **Sequences of probes(5'-3')** |
| --- | --- |
| **RP11-594N15.3** | CTTTTTGGACTGAAGAACGTGC |
|  | TGAACTTAAGTATTTACATCCC |
|  | TGTTTTATTCTTGCGATCTGAG |
|  | TTATTTCACTGGTTAGCATTTT |
|  | AATGAGTAGAGACACTAGAGGC |
