# Supplementary material for: Construction of competing endogenous RNA interaction network as prognostic markers in metastatic melanoma
Source: PeerJ. 2021 Sep 15;9:e12143. doi: 10.7717/peerj.12143 (PMC8449535; doi:10.7717/peerj.12143)
Supplement: Supplemental Information 2 [file peerj-09-12143-s002.docx]

**Supplementary table 2. Sequences of plasmids and siRNA**

| **miRNA-has-mir-3662** | target sequence | GAAAATGATGAGTAGTGACTGATG |
| --- | --- | --- |
|  | shDNA tamplate sequence | **S** 5'**-**AATTCGAAAATGATGAGTAGTGACTGATGGTTTTGGCCACTGACTGACCATCAGTCTACTCATCATTTTCA-3' |
|  |  | **A** 5'**-**CCGGTGAAAATGATGAGTAGACTGATGGTCAGTCAGTGGCCAAAACCATCAGTCACTACTCATCATTTTCG-3' |
| **miRNA-ShNC** | target sequence | AAATGTACTGCGCGTGGAGC |
|  | shDNA tamplate sequence | **S** 5'-AATTCGAAATGTACTGCGCGTGGAGACGTTTTGGCCACTGACTGACGTCTCCACGCAGTACATTTCA-3' |
|  |  | **A** 5'-CCGGTGAAATGTACTGCGTGGAGACGTCAGTCAGTGGCCAAAACGTCTCCACGCGCAGTACATTTCG-3' |
| **LncRNA-RP11-594N15.3** | target sequence | CACAGGCAGTAATGACCAAACAGAGACCAAGGTGGAGCTCAAGAGAAAGTAAGCATCAAACAGCACTCAGCCTGAATCCAGGCACGTTCTTCAGTCCAAAAAGCAAAGGGGATGTAAATACTTAAGTTCATCTTACTCAGATCGCAAGAATAAAACACCACATAGGGACTTGGGACCAACAAAATGCTAACCAGTGAAATAAGTTGTAGAGCCTCTAGTGTCTCTACTCATTGTGTATTTCTTGTATCTGTCTAATTCAGGTGTGGGGATTCAGGATCCTTTTCATCTTTAATACTAAAGTCAAAATTAACAAAATTATATGACTATAACCTATTAACTTTCTAATTTGCACCACATCTTCTTTCTGATTGGGTTAATATGGGTGCTTCCTCAGCAACCTTCCTGCTAGTCTGGACTCTATTGTTCATCTTTCTCCTAGGTGCTCCTCAGGTTCCACATTTTCTTGCCTTTCTGCCATCTCTGCCCCATCAAAGTCAATTAGGCCACTCACTTCTCTTTACTTGTCTTCTGGATGACTGTAAAAAGTTTAAAACAATGCTTATTCATTGTCTGCAACCTCAATCTAGACATAAGTGCTGCACAGCTCTCTCTTTTCATGTCCCTGGGGACAGAGGAATCTTCCCACTTTCAGGCTAAGATTTATTCCTTCATTTGATGTTTTTAAACCACTTTATTCTGACTTTAACAGGATGTCTTAATTATCCTTTTTCTTTACTGACTTTACCCTTTTTCACAAATGTGCTCAAGTTTTCAATTTCCCATTTGCTTCATCACTTCTGTTTCTCAAAATAGTAATGTATACTCCCTATTTTCATGCTTCTTCTCCTCCTTATTTCTTATTCTTTTGCAATTTGGCTTCCAATAAAATTTTTCTCATTATCAATGACATCTTTAGTAGCTCATCGTCTCTGCCATAGTCATTGAATCTCTGACCAATGTGGAAATTAAAAGGTGATGATGATTCAGAGGACTCAGATTAACCTAAAATAAAATTTAATGTTTTTATATTTTTAATTGATACTAGTATGACAGAGTATCACTGAATAATATAGTATTTTTCAACTATATAGAATCGACATATATCAAGAAAACGTCAGCTTTCTCTCTAGTATAGAACTTCCATAATGTATGGATATGTCCTTGCTCTATGACCACAGTCTCCCCGTAATGTTTGCTTGCTATGTACTCAGATCCTATAGTGCTCATTTCTTTATAGGAGTGGTTGTCACATATCTGGGCACCAACACAGAGCCTAGACATTCATAGATGGGCAAGATTACTTTGTGATGTTGTAAAGTTCTGTGTTCCATTCTACTCTTGGATAAATGTTAGTGATATCACTTGTTCCTCTCAACAGCAGCCATATATGTGTAAAATCCCAGTCTTGCTCTACTTCTCTGAATTATACCTAAACAAATATATCTACCCGAAATAGAGTGGATCATTTTTGTATATTTTATTAGCTTACTCAATGACATTTAATCTGGTTCACCATGGAATCTTTTTGATATGCTTCATTTGCCTTCCTTTTTCTTCCAGATGTTTTTTATTTAGTGAGTTCTTGTTTGTTTCTCTCTGACACCTCCTCACTAACCTAAGACTGGTTCCTCCTTGTCTACCTCTCCACTAAATTTCATGGTTCCCTGGGTTTCCATTCTCATTCCTCTCCTCTAATCTCTCTCTCCAGATGACTTCCTCCACTCCCACATCAGCAACTATATTCCTATGGCGAATCTCATATCTTGATCTCTAGCCCAGGAGTCACTCCTCATTATCTGACCTATTGGATAGTTGAACATGGATTGCTCGAAGAATCTTAAAATTCAATGTTCTAATACTAAATCCAATGCATAATCCCATTTTCAAACCTGCTTTTCCTTATATGCTACAGATTTCAGATCATGGTAGCACTGATTTTCCACTGACCTTAAATCAAAATTTTTGGAGCCCTTTTAGCTGTTACCTCTTCCTCTGATTTGTGTGTTTGTTTGTTTTTTGGTCAATCGATTCTAATTCTTCACTACTGAAACTGTTCACTTCTTTTTATCTGCACTCCAACCCTTAAATCATTACTTATCTATTATTATGCAATGGCCTGCTCAGCATGACCTGCCTCATCCAGCACTCTTTCTAGCCATCTTGCCACCAGATGTATTGCTCTGATACATGATTTTGATCATGTATCTCTCCTAATTAAAAAATCCTTAAGTTTAGCCAGACAAACTTTACGGTATAAGTACCACATTGTATTTGTGTATAACTCCTTGCTCTGTGTTAGCTTTTATCCTGTTCCTAAAATGTGCTTCATGCTTGTGGGCAAAGTCTCATTGATCCTTTCAGACTAAGCCTCATTTCATCCTTTTCGGTAGTCTTTCTGACTCATCTCCATTCCCGAGCCCTACCCTAGGAAATCAGCCATTCTATACTCCTTGCTCTCTAACAATTTATCCACAAATGGATCTCTATATCATAGCTATTTATTTGCATGCATATCTCTCCACCTTGAGTGTGAGCTTTTTGAGGGCATCAACTCCGTTTTATTCATCTTTGTATACTCACTGTTTATCTATGTAGGTTATTGCTAATCAGTACAAACATCAGTAGATATTTGTAAATAAATAATATCTGCCCTCCTGAAGCTCATAATGAAGTTAAGAGAGAGAATCTACACATAGATCGATTAGGGAAAATGCAATTCAGAGATAAAATTCATGGTTGAAAATAGTGACGTCTCAAGAATATTGCATTGCAGAGGATAAAAACTTAATTCCTATCACCATTTTTTTTAGTTTATACAAGAGTAACTATTTTAAAATTTCTATTGCAAACTGGTGTGCTGCATTTGTACACACACACACACACACACACGCACAAAGATTTGATAAGACATAGAGAAATGAGATGTCCAATAGGACAACACGTAACTAGATGGGCAAATTAAGGTAAGCCATCCAGCTAAATAAGCCTTTGGTAAATTTATTTCTGGGAATTCTGTAGCAGGAATTATCTTCAGTTTGCTGGAATTAAGATGAGCCAAAAGGGGGTTATATCTTACTCTTTTGGCCTAAGCAATACATTGTTCAGAGAGGAGGATCTCCAGGAAGGATCTTGCAGGCCTTGAACAAAAGTTCACTGGAGTTTCTGAGAATATGCCTTACAGAAATGCACTCAAACAGCCTAAAGAAATGAGAGTTCTAATAACAATAGAATAAACCTCAGTGTCAGGAATTCACAGAAAAAAGGGGGAAAAATTGTAGAAAGGGATTAAGAAACACATTTTTATATACTCAAGAAAGAAAGCTCACTGATATACATGTATAGAACCTTAAAGACCTTTTCCTGAATAGTGTTATCTTTTAAAAAAACACTGGTAATAAAATTAACTTTGGAGATCTAACAAGACTAAAGAAGTACTGTGAACTTTTGGACTCTGAAACTAAGAAAAAGACTGACAAAGGTGATGATGTAGGTTACTGCTAATAGAACATGTTTAAACTTTGGCTGCCATGATTCATTTTACACCTTGTTTGAGTAGGATGGTGTGACTATTATGACTGTCCTGTTGAATTTCTAAGTTTTCTTTTATCCCATATCCTCTTTGAAATTCACAAGCCAAGATATAATCATGAAGTTTTAAGGGTAAAAAAACCTATTCTGCCTTCTCAGATAGTCCTAAATACAATTCAACAAATCATCAAATTTGTCTAGGAAATAAAATTTGAGCATTTACTAA |
|  | siRP11-594N15.3 | **S** 5'-CCCUCCUGAAGCUCAUAAUTT-3' |
|  |  | **A** 5'-AUUAUGAGCUUCAGGAGGGTT-3' |
|  | si-NC | **S** 5'-UUCUCCGAACGUGUCACGUTT-3' |
|  |  | **A** 5'-ACGUGACACGUUCGGAGAATT-3' |
