# Supplementary material for: Construction of competing endogenous RNA interaction network as prognostic markers in metastatic melanoma
Source: PeerJ. 2021 Sep 15;9:e12143. doi: 10.7717/peerj.12143 (PMC8449535; doi:10.7717/peerj.12143)
Supplement: Supplemental Information 3 [file peerj-09-12143-s003.docx]

**Supplementary table 3. Primer sequences for quantitative real-time PCR**

| Primer Name | Primer sequences |
| --- | --- |
| miR-3662-forword | GAAAATGATGAGTAGTGACTGATG |
| miR-3662-reverse | GCGAGCACAGAATTAATACGAC |
| U6-forword | CGCTTCGGCAGCACATATACTA |
| U6-reverse | CGCTTCACGAATTTGCGTGTCA |
| RP11-594N15.3- forword | CCACATAGGGACTTGGGACC |
| RP11-594N15.3- reverse | TCCTGAATCCCCACACCTGA |
| PKIA-forword | GCCTTGAAATTAGCAGGTCTTGA |
| PKIA-reverse | GCTTCCCCACTTTGTTCTGTAG |
| CSF2RB-forword | AGCGGCTTCAGGACTCTTG |
| CSF2RB-reverse | CTGGGCATGAGGTGCTCTG |
| ZNF831-forword | CAACCCTGGCGTAAGTTGC |
| ZNF831-reverse | GAGTCGGTGCTCTCACACT |
| GAPDH-forword | AAGGTGAAGGTCGGAGTCAA |
| GAPDH-reverse | AATGAAGGGGTCATTGATGG |
