# Supplementary material for: Construction of competing endogenous RNA interaction network as prognostic markers in metastatic melanoma
Source: PeerJ. 2021 Sep 15;9:e12143. doi: 10.7717/peerj.12143 (PMC8449535; doi:10.7717/peerj.12143)
Supplement: Supplemental Information 4 [file peerj-09-12143-s004.docx]

**Supplementary table 4. Clinical characteristics of MM patients**

| **Clinical parameters** | **Whole cohort**  **(N=470)** | **In situ samples**  **(n=103)** | **Metastatic sanples**  **(n=367)** |
| --- | --- | --- | --- |
| Gender, n(%) | | | |
| Male | 291(62) | 61(59) | 230(63) |
| Female | 179(38) | 42(41) | 137(37) |
| Age | | | |
| Mean(SD) | 58.2(15.7) | 64.7(14.0) | 56.3(15.7) |
| Median[Min, Max] | 58[15, 90] | 65[23, 90] | 56[15, 87] |
| Race, n(%) | | | |
| Asian | 12(2.6) | 7(6.8) | 5(1.4) |
| White | 447(95.1) | 94(91.3) | 353(96.2) |
| Black or African American | 1(0.2) | 0(0) | 1(0.2) |
| NA | 10(2.1) | 2(1.9) | 8(2.2) |
