# Supplementary material for: Construction of competing endogenous RNA interaction network as prognostic markers in metastatic melanoma
Source: PeerJ. 2021 Sep 15;9:e12143. doi: 10.7717/peerj.12143 (PMC8449535; doi:10.7717/peerj.12143)
Supplement: Supplemental Information 5 [file peerj-09-12143-s005.docx]

**Supplementary table 5. TNM staging of AJCC pathology in MM patients**

| **TNM classification** | **Whole cohort(N=470)** | **Alive(n=246)** | **Dead(n=223)** | **NA(n=1)** |
| --- | --- | --- | --- | --- |
| T0 | 23 | 15 | 8 | - |
| T1 | 10 | 7 | 3 | - |
| T1a | 22 | 13 | 8 | 1 |
| T1b | 10 | 6 | 4 | - |
| T2 | 32 | 15 | 17 | - |
| T2a | 31 | 12 | 19 | - |
| T2b | 15 | 10 | 5 | - |
| T3 | 14 | 7 | 7 | - |
| T3a | 39 | 17 | 22 | - |
| T3b | 37 | 16 | 21 | - |
| T4 | 15 | 8 | 7 | - |
| T4a | 25 | 14 | 11 | - |
| T4b | 113 | 62 | 51 | - |
| Tis | 8 | 3 | 5 | - |
| Tx | 47 | 27 | 20 | - |
| NA | 29 | 14 | 15 | - |
| N0 | 235 | 123 | 112 | - |
| N1 | 17 | 9 | 8 | - |
| N1 | 19 | 7 | 12 | - |
| N1 | 38 | 21 | 17 | - |
| N2 | 6 | 4 | 2 | - |
| N2 | 13 | 7 | 6 | - |
| N2 | 21 | 11 | 10 | - |
| N2 | 9 | 3 | 6 | - |
| N3 | 55 | 28 | 27 | - |
| Nx | 36 | 22 | 14 | - |
| NA | 21 | 12 | 9 | - |
| M0 | 418 | 215 | 203 | - |
| M1 | 5 | 3 | 2 | - |
| M1a | 4 | 2 | 2 | - |
| M1b | 5 | 3 | 2 | - |
| M1c | 10 | 6 | 4 | - |
| NA | 28 | 17 | 11 | - |
