# Supplementary material for: Construction of competing endogenous RNA interaction network as prognostic markers in metastatic melanoma
Source: PeerJ. 2021 Sep 15;9:e12143. doi: 10.7717/peerj.12143 (PMC8449535; doi:10.7717/peerj.12143)
Supplement: Supplemental Information 6 [file peerj-09-12143-s006.docx]

**Supplementary table 6. Identified lncRNA and mRNA co-expression modules and the number of genes its contained**

| Module | black | blue | brown | green | gery | magenta | pink | red | turquoise | yellow |
| --- | --- | --- | --- | --- | --- | --- | --- | --- | --- | --- |
| lncRNA | 163 | 784 | 291 | 206 | 1910 | 44 | 143 | 201 | 1031 | 227 |
| mRNA | 110 | 1016 | 565 | 190 | 1247 | - | 38 | 143 | 1255 | 436 |
