# Supplementary figures and images for: Construction of competing endogenous RNA interaction network as prognostic markers in metastatic melanoma
Source: PeerJ. 2021 Sep 15;9:e12143. doi: 10.7717/peerj.12143 (PMC8449535; doi:10.7717/peerj.12143)

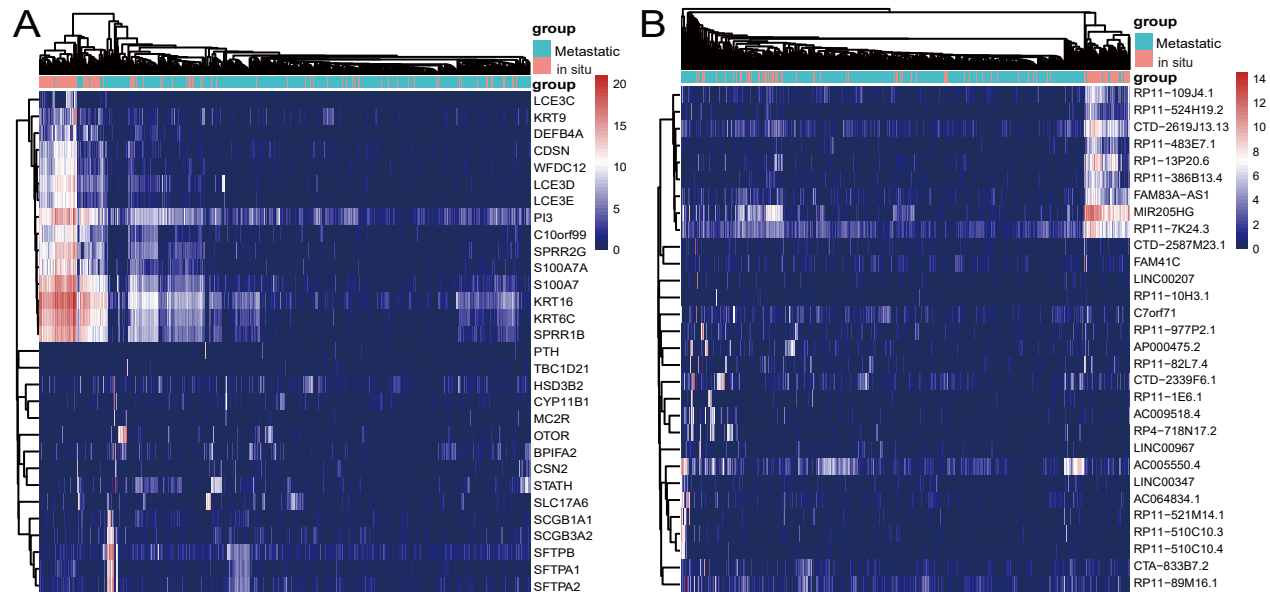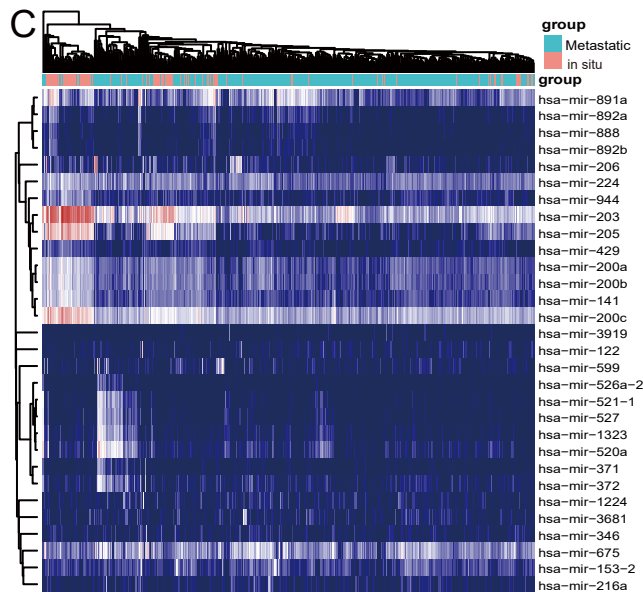

Supplement: Supplemental Information 7 — (A) Clustering heatmap demonstrate the top 15 DElncRNAs of high expressed in the in situ group and metastatic group respectively. (B) Clustering heatmap demonstrate the top 15 DEmRNAs of high expressed in the in situ group and metastatic group respectively. (C) Clustering heatmap demonstrate the top 15 DEmiRNAs of high expressed in the in situ group and metastatic group respectively. [file peerj-09-12143-s007.pdf]

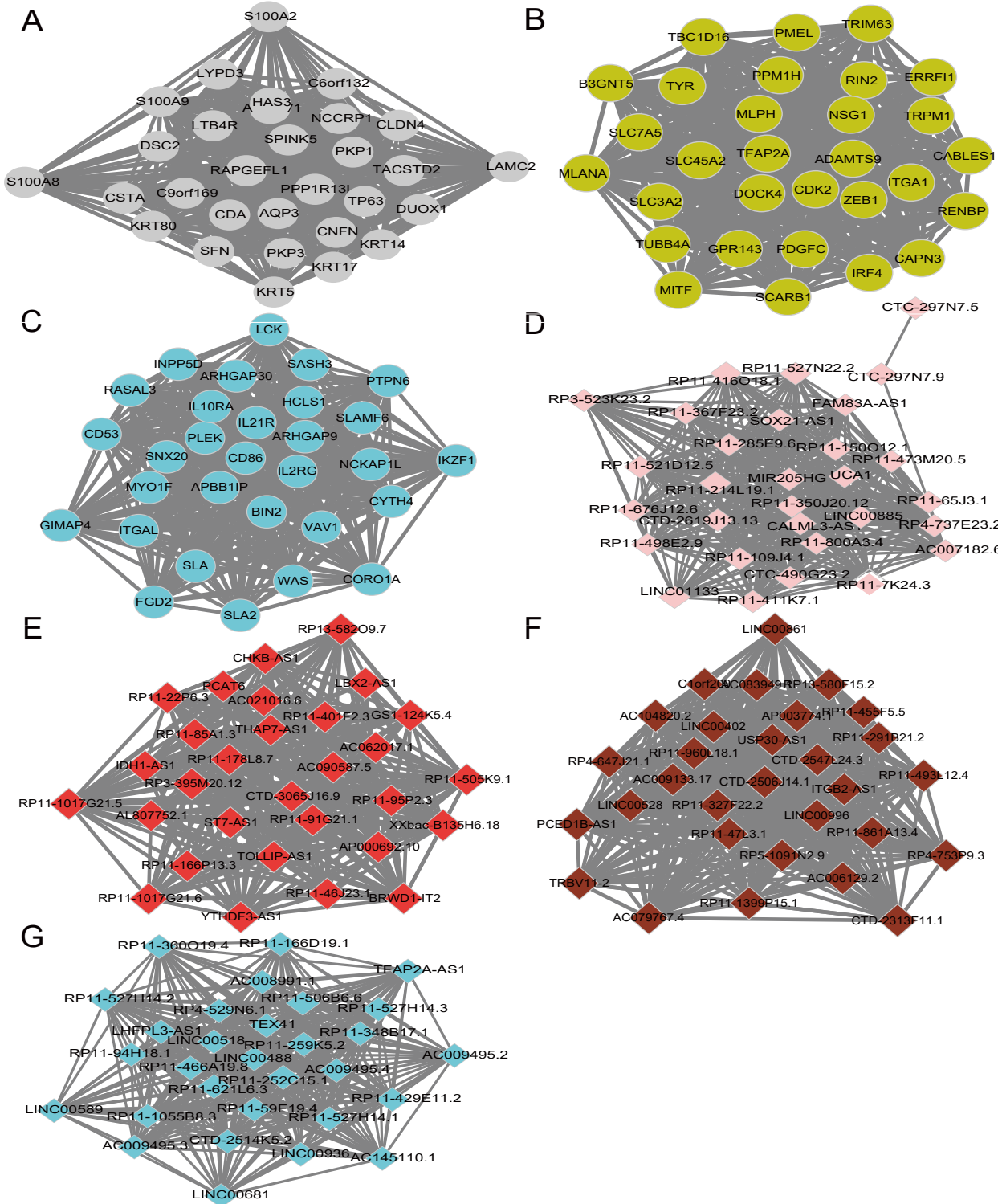

Supplement: Supplemental Information 8 — (A-C) Co-expression network of 30 most connected mRNA in the black,yellow and turquoise module. (D-G) Co-expression network of 30 most connected mRNA in the pink,red,brown and turquoise module. [file peerj-09-12143-s008.pdf]

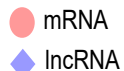

Supplement: Supplemental Information 9 — Diamond nodes represent DElncRNAs, circle nodes represent DEmRNAs. [file peerj-09-12143-s009.pdf]
